# Accumulation of small peritoneal macrophages and dendritic cells in a concomitant immunity model associated with prolonged survival

**DOI:** 10.3389/fimmu.2026.1802971

**Published:** 2026-07-23

**Authors:** Milda Vanagaitė-Žičkienė, Jurgita Juršėnaitė, Mantas Radzevičius, Rėda Matuzevičienė, Vytautas Kašėta, Rimantas Eidukevičius, Dainius Characiejus

**Affiliations:** 1 State Research Institute Centre for Innovative Medicine, Vilnius, Lithuania; 2 Vilnius University, Faculty of Medicine, Institute of Biomedical Sciences, Vilnius, Lithuania; 3 Faculty of Health Care, Vilniaus Kolegija/University of Applied Sciences, Vilnius, Lithuania; 4 Centre for Laboratory Medicine, Vilnius University Hospital Santaros Clinics, Vilnius, Lithuania; 5 Vilnius University, Faculty of Mathematics and Informatics, Vilnius, Lithuania

**Keywords:** concomitant tumor immunity, dendritic cells, SL2 lymphoma, small peritoneal macrophages, survival

## Abstract

**Purpose:**

This study investigated the contribution of peritoneal macrophages to tumor control using a DBA/2-SL2 murine model of concomitant tumor immunity.

**Methods:**

Female DBA/2 mice were subjected to a concomitant tumor model in which SL2 lymphoma cells were injected subcutaneously (SC) to establish a primary tumor, followed by intraperitoneal (IP) SL2 challenge to induce secondary tumors. Peritoneal immune cells were analyzed by flow cytometry 4 days after IP challenge. Absolute cell numbers were calculated from total viable cell recovery. To assess functional antitumor activity, CD11b^+^F4/80^+^ peritoneal macrophages were isolated by magnetic separation and adoptively transferred into naïve mice together with SL2 cells. In additional experiments, mice received repeated IP injections of SL2 cells. Non-parametric statistical tests were used for data analysis.

**Results:**

Prolonged survival in mice with secondary IP SL2 tumors was associated with pronounced alterations in peritoneal immune composition. Flow cytometric analysis revealed a marked accumulation of small peritoneal macrophages (SPMs) and dendritic cells (DCs) in mice with secondary IP tumors. Absolute counts of SPMs (CD11b^int^F4/80^int^) and DCs (CD11c^+^) were significantly increased in the secondary IP tumor group, with a positive, not statistically significant, correlation between these population. In contrast, mice with primary IP tumors showed expansion of CD11c^-^CD11b^-^ F4/80^-^ cells, which may include SL2 tumor cells, reduced numbers of B lymphocytes (CD19^+^) and large peritoneal macrophages (LPMs) (CD11b^hi^F4/80^hi^) and an increased number of CD11b^low^F4/80^int^ cells. Adoptive transfer of peritoneal macrophages from mice with secondary IP tumors did not prolong survival. Repeated IP administration of SL2 cells was associated with improved survival in mice bearing SC tumors in 3 of 5 mice, compared with 1 of 5 mice in the SC tumor-only group, although this difference did not reach statistical significance.

**Conclusion:**

Prolonged survival of DBA/2 mice with secondary IP SL2 tumors is associated with a distinct peritoneal immune landscape characterized by accumulation of SPMs and DCs. While macrophages alone were insufficient to control tumor growth, repeated IP tumor challenge suggested a trend toward systemic antitumor activity. Collectively, these findings highlight SPMs and DCs as prominent cells associated with survival benefit in this model.

## 1 Introduction

Tumor-associated macrophages (TAMs) have emerged as key regulators of tumor biology, both inhibiting and promoting malignancy (1). Infiltration of tumors by macrophages has been associated with both poor (2, 3) and favorable (4, 5) patient outcome. The apparent dual role of TAMs has been attributed to polarization of these cells into pro-inflammatory M1 (CD80^+^, CD86^+^, MHC II^+^ and CD64^+^) and anti-inflammatory M2 (CD163^+^, CD204^+^, CD206^+^, and FIZZ1^+^) phenotypes (6, 7). However, the evidence is accumulating that the M1/M2 model of macrophage polarization may be an oversimplification. CD80, a marker of M1 macrophages, was found to be a potent worse prognostic factor in patients with non-small cell lung carcinoma (2). It has been found in gastric cancer that macrophage marker expression differs between tumor areas, reflecting the coexistence of a spectrum of TAMs populations (8). Thus, it remains unclear which macrophage subpopulations and how may be exploited in cancer immunotherapy.

Previously we have studied the mechanisms of tumor rejection in the syngeneic murine DBA/2-SL2 concomitant immunity system. In this system, 10^4^ SL2 tumor cells injected subcutaneously (SC) can kill DBA/2 mice within 30 days. However, SL2 tumor cells given several days after the first tumor cell injection do not develop into a tumor. This indicates that SL2 tumor cells are killed at the site of the secondary tumor challenge. Both primary tumor implants (which are not rejected) and secondary tumor implants (which are rejected) contain 40-50% macrophages which are cytotoxic *in vitro* to SL2 tumor cells (9).

In the present study, we induced the primary SL2 tumor in DBA/2 mice SC and the secondary SL2 tumor intraperitoneally (IP). The peritoneal cavity in mice is a unique compartment to study the interaction of macrophages and tumor cells. Approximately 40% of the murine peritoneal cavity cells are CD19^+^ B lymphocytes. Among the remaining “non-B” peritoneal cavity cells, the majority are macrophages coexpressing CD11b and F4/80 markers. Relatively recently two distinct populations of macrophages have been identified: large peritoneal macrophages (LPMs) and small peritoneal macrophages (SPMs) (10, 11).

LPMs dominate the steady-state peritoneal cavity and express high levels of F4/80 and CD11b with comparatively low MHC II expression. They arise predominantly from embryonic precursors and depend on tissue-derived signals, including the transcription factor GATA6, for their maintenance and niche-specific functions. LPMs contribute to homeostasis by supporting IgA production by peritoneal B-1 cells and by functioning as resident sentinel cells capable of rapid aggregation and efferocytosis following perturbation (11, 12). By contrast, SPMs express lower levels of F4/80 and CD11b but high levels of MHC II. They originate largely from bone-marrow–derived monocytes that enter the peritoneal cavity in response to inflammatory stimuli. SPMs expand dramatically during infection or inflammation when LPMs undergo the macrophage disappearance reaction (10, 11).

Although less abundant than macrophages, peritoneal CD11c^+^ MHC II^+^ dendritic cells (DCs) represent another critical innate immune population. As professional antigen-presenting cells, they bridge innate and adaptive immunity by capturing antigens, migrating to lymph nodes, and priming T cells for pathogen clearance or tolerance. Unlike phagocytic LPMs/SPMs, DCs orchestrate Th1/Th2 responses, suppress excessive inflammation, and modulate homeostasis in conditions like peritonitis or endometriosis. Their dysregulation promotes disease progression, highlighting therapeutic potential (13).

Taken together, the peritoneal cavity contains a highly specialized innate immune ecosystem in which LPMs, SPMs, and DCs coexist and respond dynamically to perturbations, infections, or tumor challenge. Understanding the relative contributions of these populations to antitumor immunity is therefore crucial. The aim of the present study was to assess the role of peritoneal macrophages in the control of SL2 tumor growth in DBA/2 mice.

## 2 Materials and methods

### 2.1 Laboratory animals

Female DBA/2 mice (8–10 weeks old) were used in this study. The DBA/2 mice were bred and kept under controlled conditions at the animal housing facility of State Research Institute Centre for Innovative Medicine, Vilnius, Lithuania. The mice were housed in individually ventilated cages under a 12/12 h light/dark cycle, at 20-24 °C and 40–60% humidity to ensure their welfare throughout the study period. The mice had free access to food and water. Mice were euthanized by CO_2_ inhalation prior to peritoneal cell collection. All procedures were performed in accordance with national and institutional guidelines for the care and use of laboratory animals. All experiments were approved by the Lithuanian State Food and Veterinary Service (approval No. G2-211).

### 2.2 Tumor

The SL2 lymphoma originated spontaneously as a peritoneal ascitic tumor in a DBA/2 mouse at the Chester Beatty Research Institute now the Institute of Cancer Research (ICR) in the laboratory of Dr. P. Alexander (14). The Department of Pathology at Utrecht University subsequently obtained SL2 cells through Prof. W. Den Otter (15), who later kindly provided the cells to one of the authors of this study (D.Ch.). SL2 is a murine T cell lymphoma expressing T cell markers and is readily transplantable, growing in both solid and ascitic forms (16).

SL2 lymphoma cells were cultured in RPMI-1640 medium (Genaxxon Bioscience) supplemented with 10% fetal bovine serum (Thermo Fisher Scientific), L-glutamine, and penicillin/streptomycin (Genaxxon Bioscience). Cultures were maintained at 37 °C in a humidified environment containing 5% CO_2_ and cell viability was determined using trypan blue (Gibco). To obtain sufficient numbers for subsequent experiments SL2 cells were further amplified *in vivo* by IP injection into a DBA/2 mouse.

One week after the IP injection of SL2 cells into a donor mouse, ascitic SL2 cells were harvested from the peritoneal cavity and washed twice with sterile phosphate-buffered saline (PBS) (Genaxxon Bioscience). SC tumors were induced by injection of 1 × 10^6^ or 1 × 10^4^ SL2 cells in 100 μL PBS and IP tumors were induced by injection of the same amount of SL2 cells in 1 mL PBS. Five mice were used in each experimental group, unless otherwise specified in the figure legends. Mice were regularly monitored for signs of tumor progression, including ascites development.

Peritoneal cells were harvested by IP lavage with 5 mL of ice-cold PBS. Following gentle abdominal massage, peritoneal fluid was aspirated using a 5 mL syringe fitted with a 21-gauge needle. Cells were pelleted by centrifugation at 2, 000 rpm for 5 min at 4 °C and resuspended in ice-cold PBS. Total viable cell numbers were determined using a Neubauer hemocytometer (Carl Roth) under a light microscope (Olympus).

### 2.3 Flow cytometry of peritoneal cells

For flow cytometric analysis, 1.5 × 10^6^ cells per sample were stained with a Fixable Viability Dye eFluor™ 506 (BioLegend) in PBS for 15 min. at room temperature, protected from light. Fc receptors were blocked using anti-mouse FcγRIII/II antibody (clone 2.4G2; BioLegend). Cells were stained for 30 min. at 4 °C in the dark using the following fluorochrome-conjugated antibodies: CD19 (PerCP-Cy5.5), F4/80 (FITC), CD11b (APC), CD11c (PE) (all from Cell Signaling Technology). Data were collected using a FACSCanto II flow cytometer (BD Biosciences) and analyzed using FACSDiva software v8.0.2 (BD Biosciences). Peritoneal immune cell subsets were identified and quantified using sequential gating, according to the strategy described by Bou Ghosn et al. (10).

The total number of peritoneal cells recovered per mouse was calculated by multiplying the cell concentration by the total volume of peritoneal lavage fluid collected. Absolute numbers of individual cell populations were calculated as follows: absolute cell number = total viable peritoneal cells recovered × (% of subset/100).

### 2.4 Transfer of peritoneal macrophages

To evaluate the antitumor activity of activated IP macrophages, an adoptive transfer experiment was performed. Peritoneal cells were collected from donor mice on day 4 after secondary IP tumor implantation and resuspended in PBS supplemented with 0.5% bovine serum albumin (Sigma-Aldrich) and 2 mM EDTA (Millipore). Macrophages were isolated using the Macrophage Isolation Kit (Peritoneum), mouse (Militenyi Biotec) according to the manufacturer’s protocol. Briefly, non-macrophage cells were magnetically labeled with a biotin-conjugated antibody cocktail and anti-biotin MicroBeads (both included in the kit). The labeled cells were retained on MS columns inserted into a MiniMACS separator (Militenyi Biotec), and unlabeled F4/80^+^ macrophages were collected in the flow-through fraction. After isolation, 8 × 10^5^ cells collected from the effluent were transferred to the peritoneal cavity of intact mice together with 8 × 10^5^ SL2 cells. Control mice received only 8 × 10^5^ SL2 cells. Cells were kept on ice throughout the procedure until IP transfer. After transfer, mice were monitored daily for tumor progression and survival was recorded.

### 2.5 Statistical analysis

Comparisons among three groups of cell subset proportions were analyzed using the Kruskal-Wallis test. When a significant overall difference was detected, pairwise group comparisons were performed using Dunn *post-hoc* test with adjustment for multiple comparisons. Comparisons of absolute cell numbers between two groups were performed using the nonparametric Mann-Whitney U test (Wilcoxon rank-sum test). Correlation between SPM and dendritic cell (DC) counts was assessed using Spearman’s rank correlation analysis. Survival data were analyzed using Kaplan-Meier survival analysis and the log-rank (Mantel-Cox) test. A p-value < 0.05 was considered statistically significant. All statistical analyses were performed and graphs were created using GraphPad Prism version 10.5.0 (GraphPad Software).

## 3 Results

### 3.1 Prolonged survival of mice with secondary IP tumors

IP tumors are generally known to cause more rapid mortality in mice than SC tumors. This is likely because IP tumors can disseminate more readily throughout the peritoneal cavity, whereas SC tumors remain localized beneath the skin. The DBA/2-SL2 tumor model represents a system of concomitant tumor immunity, in which the growth of a secondary tumor is inhibited by the presence of a primary tumor (9). In this study, we sought to determine whether the growth of a secondary IP SL2 tumor in DBA/2 mice is suppressed by a primary SC SL2 tumor. If such an effect occurs, the survival of mice bearing both a primary SC and a secondary IP tumor would be expected to be longer than that of mice bearing only a primary IP tumor.

**Figure 1:**
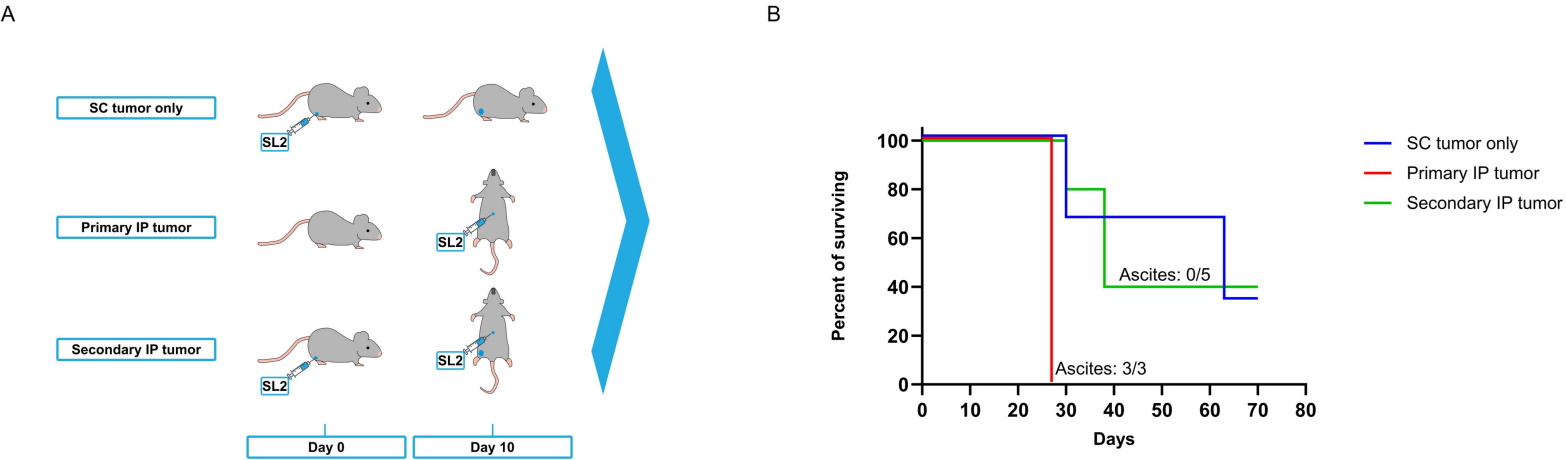
Survival of DBA/2 mice bearing IP and SC SL2 tumors. **(A)** Experimental design. Mice in the SC tumor only group (n = 3) received a SC injection of 1 × 10^6^ SL2 cells on day 0. Mice in the Primary IP tumor group (n = 3) received an IP injection of 1 × 10^6^ SL2 cells on day 10. Mice in the Secondary IP tumor group (n = 5) received an SC injection of 1 × 10^6^ SL2 cells on day 0 followed by an IP injection of 1 × 10^6^ SL2 cells on day 10. **(B)** Kaplan–Meier survival analysis. All mice in the Primary IP tumor group died by day 27, whereas 2/5 mice in the Secondary IP tumor group and 1/3 mice in the SC tumor only group survived beyond 70 days. Survival was prolonged in the Secondary IP tumor group compared with the Primary IP tumor group (log-rank test, p < 0.05), with no difference versus the SC tumor only group. No ascites formation was observed in mice bearing secondary IP SL2 tumors (0/5), whereas ascites developed in all mice in the Primary IP tumor group (3/3). SC, subcutaneous; IP, intraperitoneal.

As shown in **Figure 1**, mice that received an IP injection of SL2 cells as the primary tumor survived for 17 days. In contrast, mice injected IP with SL2 cells 10 days after SC inoculation of SL2 cells did not show ascites development. Similar findings were observed in an independent experiment (**Supplementary Figure 6**). No ascites formation was observed in mice bearing secondary IP SL2 tumors whereas ascites developed in all mice in the Primary IP tumor group.

Thus, our results indicate that the presence of a primary SC SL2 tumor is associated with improved survival in DBA/2 mice bearing secondary IP SL2 tumors.

### 3.2 Secondary IP tumor challenge drives expansion of SPMs and DCs

**Figure 2:**
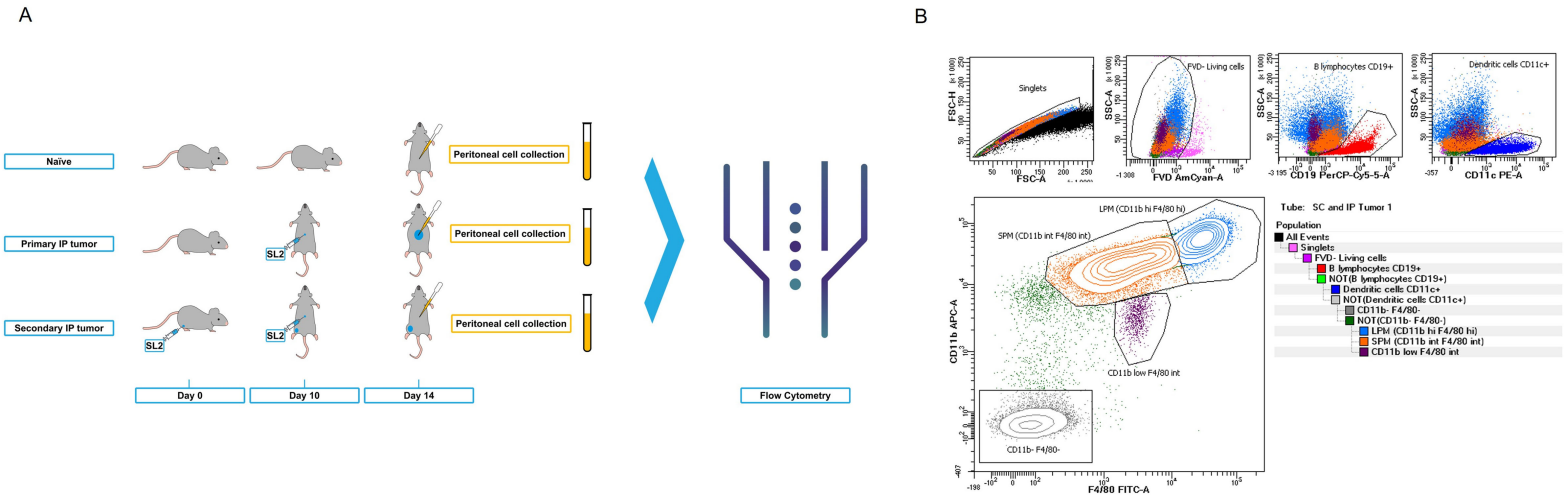
Flow cytometry of peritoneal immune cells in DBA/2 mice. **(A)** Experimental design. Mice in the Primary IP tumor group received 1 × 10^6^ SL2 cells IP on day 10 (n = 5), whereas the Secondary IP tumor group received 1 × 10^6^ SL2 cells SC on day 0 followed by 1 × 10^6^ SL2 cells IP on day 10 (n = 5). Naïve mice did not receive tumor cells (n = 4). For flow cytometric analysis, peritoneal cells were collected on day 14 by lavage with 5 mL of ice-cold PBS. **(B)** The gating strategy for flow-cytometric identification of LPMs, SPMs and other peritoneal immune cells. After exclusion of doublets (FSC-H vs. FSC-A) and non-viable cells (FVD+), CD19^+^ B cells and CD11c^+^ DCs were identified and their percentages recorded for downstream analyses. The remaining live, single CD19^-^CD11c^-^ population was subsequently evaluated for macrophage subsets based on F4/80 and CD11b expression to distinguish LPMs and SPMs. SC, subcutaneous; IP, intraperitoneal; PBS, phosphate-buffered saline; DC, dendritic cell; SPM, small peritoneal macrophage; LPM, large peritoneal macrophage; FVD, Fixable Viability Dye eFluor™ 506.

To examine differences in peritoneal immune cells between secondary and primary IP SL2 tumors, an experiment was designed in which mice were administered SL2 tumor cells following the treatment scheme presented in **Figure 2A**. This scheme delineates the timing of SC and IP SL2 cell injections. Flow cytometric immune cell gating was performed according to the strategy described by Bou Ghosn et al. (10) and is shown in **Figure 2B**. Based on this description, cells expressing lower levels of CD11b and F4/80 were considered SPMs (CD11b^int^F4/80^int^), whereas cells expressing higher levels of CD11b and F4/80 were considered LPMs (CD11b^hi^F4/80^hi)^.

**Figure 3:**
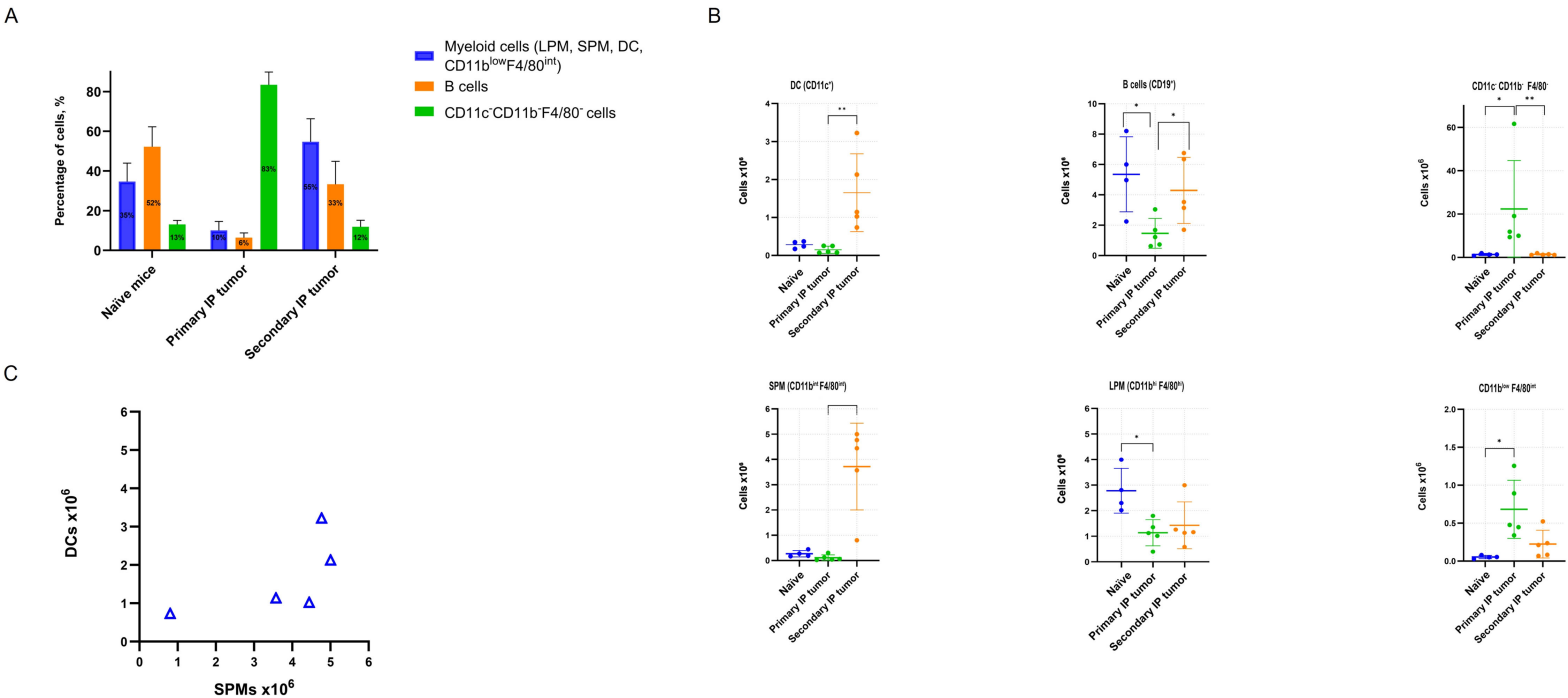
Peritoneal immune cell subsets in DBA/2 mice bearing primary or secondary SL2 tumors and in naïve mice. **(A)** Proportional composition of immune cell subsets. The experimental groups and sampling scheme correspond to those shown in **Figure 2**. Proportional analysis revealed significant differences in immune cell composition among groups (Kruskal-Wallis test). The Secondary IP tumor group exhibited a higher proportion of myeloid cells compared with the Primary IP tumor group (*p* < 0.01), whereas the Primary IP tumor group showed fewer B lymphocytes than naïve mice (p < 0.01) and more CD11b^-^F4/80^-^ cells than the Secondary IP tumor group (p < 0.05). **(B)** Absolute counts of peritoneal immune cells. The experimental groups and sampling scheme correspond to those shown in **Figure 2**. Each point represents an individual mouse; horizontal bars denote the mean ± standard deviation. Statistically significant differences between groups are indicated as follows: *p < 0.05, ** p < 0.01; Mann-Whitney U test. The experiment was performed three times with similar results; one representative experiment is shown. **(C)** Correlation between peritoneal SPMs and DCs in DBA/2 mice with the secondary IP tumors. Percentages of SPMs and DCs were determined by flow cytometry, and absolute counts were calculated as described in the Materials and methods. Each point represents an individual mouse. (Spearman r = 0.80; p = 0.13). IP, intraperitoneal; SPM, small peritoneal macrophage; LPM, large peritoneal macrophage; DC, dendritic cell.

The proportional composition of peritoneal cell subsets in DBA/2 mice bearing primary or secondary IP SL2 tumors is presented in **Figure 3A**. The Secondary IP tumor group showed the higher proportions of myeloid cells (CD11b^hi^F4/80^hi^, CD11b^int^F4/80^int^, CD11c^+^, CD11b^low^F4/80^int^) than the Primary IP tumor group. In contrast, the Primary IP tumor group showed the largest compartment of CD11b^-^F4/80^-^ cells, which may include SL2 tumor cells, compared with the Secondary IP tumor group (p < 0.05). The proportion of B cells (CD19^+^) was reduced in the Primary IP tumor group compared with naïve mice (p < 0.01).

**Figure 3B** presents the absolute counts of peritoneal immune cell subsets across the three experimental groups. Absolute counts of SPMs (CD11b^int^F4/80^int^) and DCs (CD11c^+^) were markedly elevated in the Secondary IP tumor group compared with both the Primary IP tumor group and Control mice, indicating substantial recruitment or expansion of these myeloid populations. In contrast, mice in the Primary IP tumor group displayed significantly higher numbers of other CD11c^-^CD11b^-^F4/80^-^ cells, which may include proliferating SL2 lymphoma cells. Absolute counts of B lymphocytes and LPMs were reduced in the Primary IP tumor group, whereas CD11b^low^F4/80^int^ cells (eosinophils according to Bou Gosh (10)) were increased relative to the other groups. Together, these data demonstrate that secondary IP SL2 tumor challenge elicits a pronounced recruitment or expansion of SPMs and DCs, whereas primary IP tumors are characterized by expansion of CD11c^-^CD11b^-^F4/80^-^ cells.

Because both SPMs and DCs expanded prominently in the mice rejecting the secondary IP tumor, we next examined whether their absolute numbers were quantitatively correlated. Absolute counts of SPMs and DCs showed a positive, though not statistically significant correlation consistent with their common derivation from blood monocytes (**Figure 3C**).

Taken together, our data indicates that secondary IP tumor challenge preferentially activates monocyte-derived innate populations, leading to a marked accumulation of SPMs and DCs in the peritoneal cavity.

### 3.3 Evaluation of *in vivo* antitumor effects of monocyte-derived innate cells

**Figure 4:**
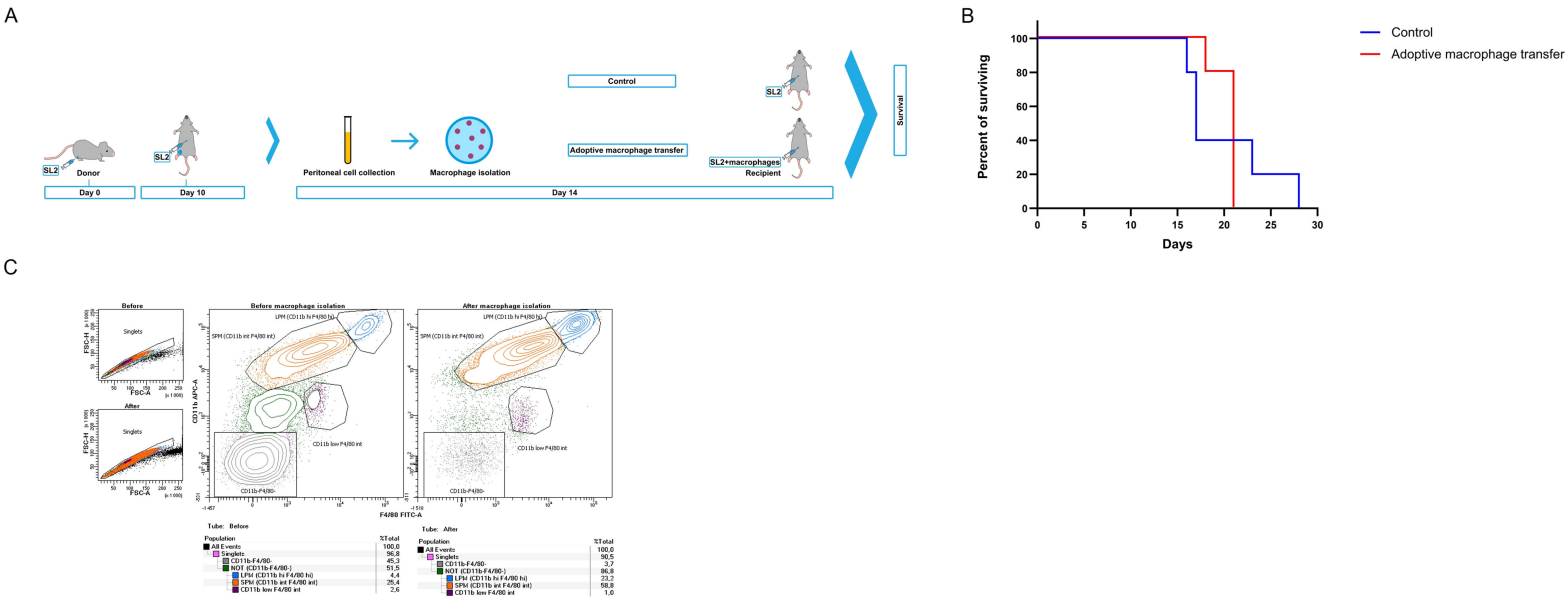
Adoptive macrophage transfer experiment. **(A)** Experimental design. Peritoneal macrophages were isolated on day 14 from donor mice (n=5) that had received 1 × 10^6^ SL2 cells SC on day 0 followed by 1 × 10^6^ SL2 cells IP on day 10. Recipient mice in the adoptive macrophage transfer group (n=5) received an IP injection of 8 × 10^5^ SL2 cells mixed with 8 × 10^5^ ex vivo-isolated peritoneal macrophages, whereas control mice (n=5) received 8 × 10^5^ SL2 lymphoma cells alone. **(B)** Kaplan–Meier survival analysis. All mice in the Adoptive macrophage transfer group succumbed by day 21, whereas control mice succumbed by day 28. Ascites formation was observed in all mice. **(C)** Flow cytometric assessment of peritoneal cell composition pre- and post-macrophage isolation. Peritoneal cells from mice that rejected secondary IP tumors were resuspended in MACS buffer (PBS containing 0.5% BSA and 2 mM EDTA) and subjected to macrophage isolation using the Macrophage Isolation Kit (Peritoneum), mouse (Miltenyi Biotec), according to the manufacturer’s instructions. Non-macrophage cells were magnetically labeled with a biotin-conjugated antibody cocktail and anti-biotin MicroBeads, leaving macrophages unlabeled in the flow-through fraction. Representative flow cytometry dot plots show CD11b and F4/80 expression before and after isolation, with CD11b^+^F4/80^+^ cells comprising 56% of singlets prior to and 96% of singlets following macrophage isolation. IP, intraperitoneal; SC, subcutaneous; PBS, phosphate-buffered saline; BSA, bovine serum albumin; EDTA, ethylenediaminetetraacetic acid; MACS, magnetic-activated cell sorting; SPM, small peritoneal macrophage; LPM, large peritoneal macrophage.

To test whether SPMs are associated with survival outcomes *in vivo*, an adoptive transfer experiment was performed. Macrophages were isolated from the peritoneal cavity of mice with secondary IP tumors (**Figure 4A**) and transferred IP into naïve recipients at the time of tumor challenge. Mice were followed to assess overall survival. As shown in **Figure 4B**, the adoptive transfer of macrophages did not significantly affect survival of recipient mice compared with control mice that received SL2 cells alone. Thus, passive transfer of macrophages alone was insufficient to prevent tumor progression under these conditions.

**Figure 5:**
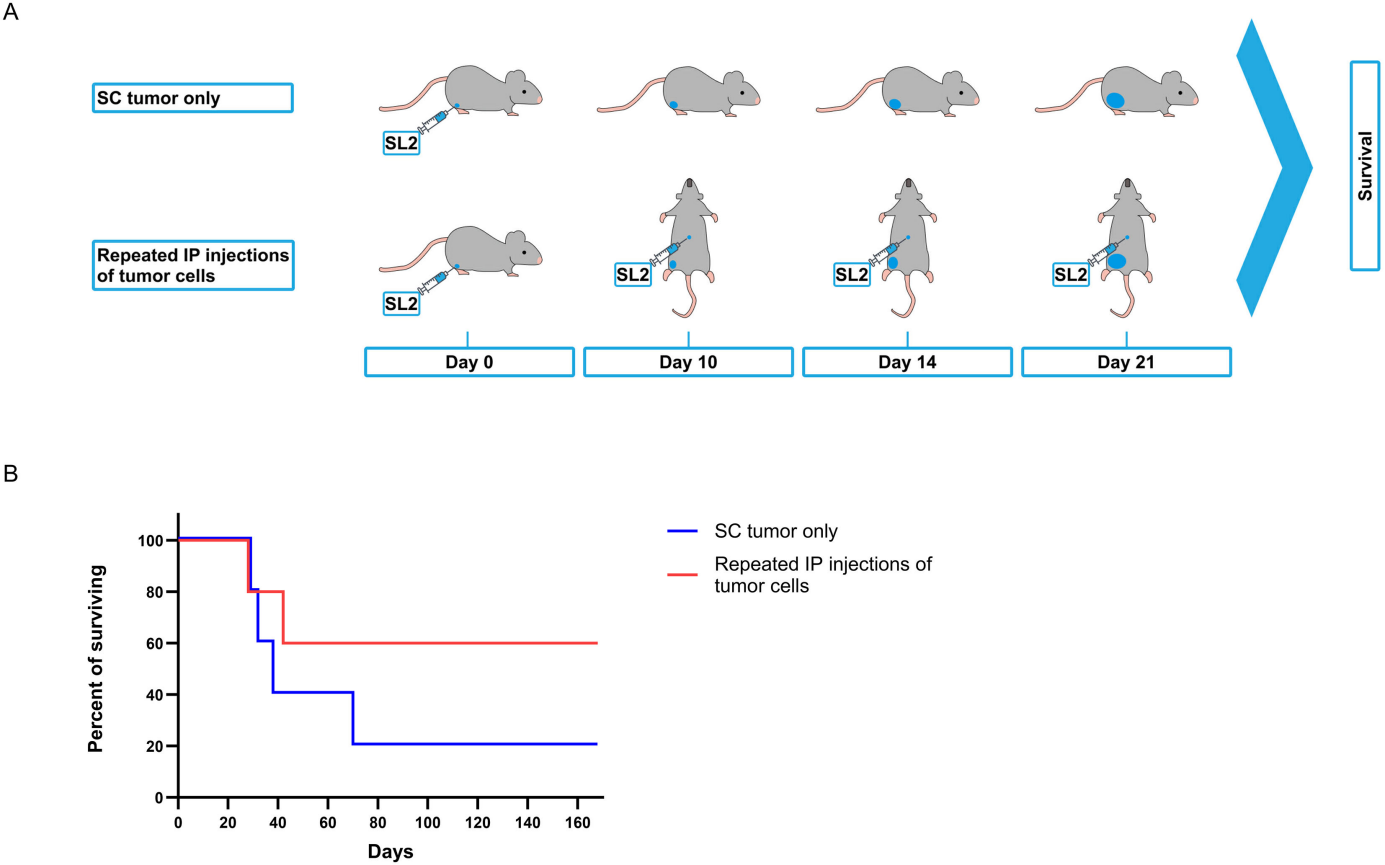
Survival of DBA/2 mice after repeated IP SL2 tumor cell injections. **(A)** Experimental design. Mice in the SC tumor only group (n = 5) received a single SC injection of 1 × 10^6^ SL2 cells on day 0. Mice in the Repeated IP injections group (n = 5) received the same SC tumor challenge on day 0, followed by IP injections of 1 × 10^6^ SL2 cells on days 10, 14, and 21. **(B)** Kaplan–Meier survival analysis. Kaplan-Meier survival curves showing 1/5 survival in the SC tumor-only group versus 3/5 in the repeated IP injections group; differences were not statistically significant by log-rank (Mantel-Cox) test (p = 0.2875). No ascites formation was observed in mice bearing secondary IP SL2 tumors (0/5). IP, intraperitoneal; SC, subcutaneous.

Next, we examined whether repeated exposure of the peritoneal compartment to SL2‐cell stimuli could modulate the recruitment, differentiation, or activation of monocytes into SPMs and monocyte-derived DCs and whether such changes might confer systemic antitumor response. The evidence of a systemic immune response would be improved survival of mice bearing primary SC tumor. In this experiment 3 out of 5 mice survived following repeated IP injections of SL2 tumor cells, compared with 1 mouse in the control group. Experimental design and survival of the mice are shown in **Figure 5** Although these differences were not statistically significant, they indicate a trend toward improved systemic tumor control after repeated IP stimulations. Similar findings were observed in an independent experiment (**Supplementary Figure 7**).

In summary, adoptive transfer of peritoneal macrophages alone did not show their antitumor activity, whereas repeated IP stimulation with SL2 cells may promote systemic antitumor activity.

## 4 Discussion

In the phenomenon of concomitant tumor immunity, the primary tumor induces an immune response, which may not be sufficient to destroy the primary tumor, but prevents the growth of a secondary tumor or metastases (17). In our experiments, we used survival as the primary indicator of tumor control, as survival is ultimately the most clinically relevant outcome for human patients. Prolongation of survival indicates tumor control. In two experiments, mice bearing secondary IP tumors (SC + IP tumors) survived significantly longer than mice with primary IP tumors alone (**Figure 1**; **Supplementary Figure 6**). Across three experiments, we also compared mice with secondary IP tumors to those bearing only SC tumors. In one experiment, survival in the SC + IP group was similar to that of the SC-only group (**Figure 1**). Notably, in two additional experiments, mice with secondary IP tumors showed a trend toward better survival than SC-only mice, although these differences did not reach statistical significance (**Figure 5**; **Supplementary Figure 7**). Taken together, the reproducible survival advantage observed in mice bearing secondary IP tumors across multiple independent experiments suggests differences in tumor progression under these experimental conditions.

Our data show that prolonged survival of DBA/2 mice with secondary IP SL2 tumors is accompanied by a marked accumulation of SPMs and DCs. While these findings suggest an association between SPM enrichment and survival outcomes in this model, they contrast with prior reports linking SPM accumulation to tumor progression. Notably, in most of these studies SPMs were analyzed at later stages of tumor development, ranging from 7 days to 6 weeks after implantation (18–21), raising the possibility that SPM function evolves over time. Although one study described rapid SPM accumulation as early as day 3 after tumor challenge with tumor-promoting effects (22).

A plausible explanation for the divergence between our findings and those reported previously lies in fundamental differences in experimental context. Previous studies have examined immune responses in primary IP tumors, whereas in our model the IP challenge was introduced as a secondary tumor in mice bearing an established SC tumor. As a result, monocyte recruitment, differentiation, and functional polarization within the peritoneal cavity are likely shaped by pre-existing systemic and tumor-driven immune conditioning, leading to macrophage responses that differ qualitatively from those observed in primary tumor settings.

In addition to the pronounced accumulation of SPMs, secondary IP SL2 tumor rejection was also associated with a concomitant increase in CD11c^+^ DCs. This coordinated expansion is biologically plausible, as both SPMs and conventional DCs can arise from circulating monocytes under inflammatory conditions (11, 23). Their parallel accumulation suggests that secondary IP tumor challenge may preferentially promote monocyte recruitment and local differentiation within the peritoneal cavity.

Emerging evidence indicates that monocyte-derived macrophages and DCs can acquire memory-like properties through epigenetic and metabolic reprogramming, a phenomenon referred to as trained immunity. Such training enables enhanced or altered innate immune responses upon secondary challenge and has been described in both macrophages and DCs following inflammatory or tumor-associated stimuli (24, 25). The concomitant immunity model in which a primary tumor confers protection against a secondary challenge offers a relevant setting to explore whether such innate memory contributes to anti-tumor responses. Our data show that secondary tumor challenge in this context triggers the accumulation of monocyte-derived macrophages and DCs. This pattern of recruitment upon re-exposure is consistent with the induction of trained immunity, suggesting that these cells may have undergone innate immune memory reprogramming. If functionally validated, this could help to better understand the mechanisms associated with the observed survival outcomes.

Macrophages isolated from secondary IP tumors did not suppress tumor growth when co−transferred with SL2 cells into naïve recipients. Our adoptive transfer experiments employed a 1:1 input ratio of 8 × 10^5^ macrophages to 8 × 10^5^ SL2 tumor cells. Although this ratio may be insufficient for macrophage-mediated antitumor activity, the absence of even slight survival benefit in our experiments argues against a meaningful direct antitumor effect of macrophages under these conditions. Adoptive transfer experiments with increased E:T ratios are needed to validate or disprove the role of cytotoxic macrophages.

We did not assess macrophage cytotoxicity in the present study because our previous work demonstrated that *in vitro* cytotoxicity does not account for the differential outcomes of SL2 tumor implantation, in which secondary implants are rejected whereas primary implants continue to grow (9). Beyond direct cytotoxicity, several alternative mechanisms may explain how macrophages inhibit the growth of secondary tumors (26).

Recent work has identified a microanatomical unit of antitumor immunity composed of macrophages, DCs, CD4^+^ T cells, and CD8^+^ T cells, underscoring the cooperative interactions required for effective immune control of tumors (27). T lymphocytes play a central role in concomitant tumor immunity. Early studies demonstrated that systemic macrophage activation arises as a consequence of T−cell–mediated immunity generated against progressively growing tumors (28). Subsequent work identified a population of Ly−1−2^+^ tumor−sensitized T cells capable of inducing the regression of established tumors when passively transferred into γ−irradiated recipients, highlighting the therapeutic potential of concomitant antitumor immunity (29). In our previous study, we found that SL2 tumors induce an expansion of effector memory T lymphocytes; however, these expanded populations were unable to inhibit tumor growth (30). Together, these findings indicate that interactions between macrophages and T lymphocytes may critically influence the efficacy of antitumor immune responses and warrant further investigation (31).

Notably, the peritoneal cavities of mice bearing secondary SL2 tumors contain a similar proportion of CD11c^-^CD11b^-^F4/80^-^ cells as those of naïve mice (**Figure 3A**), suggesting that a substantial influx of T lymphocytes into the peritoneal cavity is unlikely. Nevertheless, adoptive transfer of unfractionated peritoneal cells or nonadherent cell populations could serve as valuable comparison groups, given that T cells have been reported to function as effector cells against secondary tumors.

Taken together, our findings indicate that secondary IP tumor challenge is associated with a selective enrichment of monocyte-derived innate immune populations - most prominently SPMs and DCs - within the peritoneal cavity, supporting a context-dependent role for these cells in shaping antitumor immunity. Determining whether and how conditioned or trained macrophages functionally engage with DCs, T and B lymphocytes to shape antitumor responses is essential for leveraging macrophage plasticity in immunotherapeutic strategies.

### 4.1 Strengths and limitations

A major strength of this study is the use of a well-defined concomitant tumor immunity model that enables direct comparison of innate immune landscapes associated with different survival outcomes. Several limitations of our study must be acknowledged. SL2 lymphoma cells could not be distinguished from T lymphocytes by flow cytometry due to overlapping markers, and CFSE labeling proved insufficient for *in vivo* tracking. We acknowledge as a limitation that we transferred the entire peritoneal macrophage population including both SPMs and LPMs rather than isolating SPMs, which may possess stronger antitumor potential. Macrophage depletion was not performed, limiting functional validation of their role in tumor control and the cytotoxic activity of SPMs was not directly assessed in this study. Another limitation of this study is that the adoptive transfer experiment was performed using a 1:1 effector-to-target ratio instead of higher ratios (e.g., 10:1 or above). Despite these limitations, the study provides descriptive and comparative data on myeloid cell dynamics in a concomitant tumor immunity model, offering a foundation for future mechanistic investigations.

## Data Availability

The original contributions presented in the study are included in the article/**Supplementary Material**. Further inquiries can be directed to the corresponding author.

## Supplementary material

Supplementary Figure 6 Survival of DBA/2 mice bearing primary or secondary IP SL2 tumors. **(A)** Experimental design. Mice in the Primary IP tumor group (n = 4) received a IP injection of 1 × 10^4^ SL2 cells on day 7. Mice in the Secondary IP tumor group (n = 4) received an SC injection of 1 × 10^4^ SL2 cells on day 0 followed by an IP injection of 1 × 10^4^ SL2 cells on day 7. **(B)** Kaplan–Meier survival analysis. Survival curves show 0/4 surviving in the Primary IP tumor group compared with 1/4 surviving in the Secondary IP tumor group. Survival was significantly prolonged in the Secondary IP tumor group compared with the Primary IP tumor group by log-rank test (Mantel–Cox) test (p = 0.0084). No ascites formation was observed in mice bearing secondary IP SL2 tumors (0/4), whereas ascites developed in all mice in the Primary IP tumor group (4/4). IP, intraperitoneal; SC, subcutaneous.

Supplementary Figure 7 Survival of DBA/2 mice bearing SC and IP SL2 tumors or SC only tumors. **(A)** Experimental design. Mice in the SC tumor only group (n = 5) received a SC injection of 1 × 10^4^ SL2 cells on day 0. Mice in the SC and IP tumor group (n = 5) received an SC injection of 1 × 10^4^ SL2 cells on day 0 followed by an IP injection of 1 × 10^4^ SL2 cells on day 7. **(B)** Kaplan–Meier survival analysis. Survival curves show 1/5 surviving in the SC tumor only group compared with 4/5 surviving in the SC and IP tumor group. Although this trend indicates improved survival in mice bearing both tumors, the difference did not reach statistical significance by log-rank (Mantel–Cox) test (p = 0.0763). No ascites formation was observed in mice bearing secondary IP SL2 tumors (0/5).
